# Six Years of *KI Reports*: Milestones Behind and Ahead

**DOI:** 10.1016/j.ekir.2021.11.030

**Published:** 2021-12-06

**Authors:** Radha McLean, Sumit Mohan, Jai Radhakrishnan

**Affiliations:** 1 Division of Nephrology, Columbia University Vagelos College of Physicians and Surgeons, New York, New York, USA

*KI Reports* continues to grow with publications of clinical and translational research in kidney disease authored by an increasingly diverse group of researchers for a widespread global readership—one that benefits from the open-access model.

In the last 2 years, we experienced a dramatic increase in manuscript submissions due to both COVID-19 and non–COVID-19 areas of kidney research. The COVID-19 content of the journal has been particularly notable; some of our reports in the early stages of the pandemic included those on collapsing glomerulopathy, AKI associated with high recovery rates, and protocols for management of affected kidney transplant recipients.^1^, ^2^, ^3^, ^4^ Recent increased attention given to CKD of uncertain etiology across the globe underscores the importance of publishing such research to expand our knowledge of this devastating disease.^5^

Our ability to publish findings quickly after acceptance—a long-standing feature valued by authors—was particularly valuable for authors given the rapid pace with which our understanding of the pandemic was growing.

Despite the pandemic, we continue to aspire to meet the needs of nephrology researchers and the kidney community at large. An example of this effort is our widely popular Research Letters, which allow authors to report findings of significance in a condensed and focused manner. Our online format allows unlimited room for supplementary information so that additional methods, analysis, and graphics can be provided without sacrificing important methodological and other details. As a journal with an international reach, we also recognize the need for clinical studies on kidney disease from all over the globe, including regions with limited resources but important data to share. Our commitment to support and encourage the dissemination of these data has led to the creation of the “Regional Reports” section of the journal.

We expanded our editorial team to include Anna Francis from Brisbane, Australia, after completion of her editorial fellowship at *KI*. Sophia Ambruso from Denver, Colorado, USA, has joined the team as well. She oversees the creation of the graphical abstracts with the help of a talented team of graphical artists. Our newly improved graphical abstracts create compelling and effective visual summaries of studies. Dr. Ambruso spearheads our increased social media engagement, with the contributions of our new Twitter Editor, Gerren Hobby, some of our graphical artists, and a team of Nephrology Social Media Collective interns. This effort helps to share research while creating the opportunity for authors and readers to engage in meaningful dialogue.

Our relationship with *KI*, the flagship ISN publication, remains strong. We continue to transfer quality articles submitted to *KI* that could not be accommodated based on space limitations/priority grounds. This process ensures that quality research on kidney disease is published and benefits some authors by saving time in the submission and/or peer review process*.*

The editorial board of *KI Reports* continues to grow and evolve as we strive to reflect the geographic diversity of our authors in the board members. In 2021, we added board members from Africa, Australia, Canada, and Europe, and broadened the expertise that is represented on our Board.

Finally, none of these acheivements would have been possible without the authors who trust us with their research findings and our peer reviewers, whose commitment to providing excellent, thoughtful, and constructive reviews ensures that we continue to publish the highest quality research in *KI Reports*.

We look forward to connecting with you—our readers—and welcome comments. Feel free to e-mail us at kireports.editor@cumc.columbia.edu.

## Disclosure

JR is the Editor-in-Chief of *KI Reports*; received research grants from Travere and Bayer; is on the steering committee for Travere; and serves as a consultant/advisory board member for Aurinia Pharmaceuticals, Angion Biomedica, Akebia Pharmaceuticals, Equillium Bio, Sanofi Genzyme, Reistone Biopharma, Travere, and GlaxoSmithKline. SM is the Deputy Editor of *KI Reports* and is a consultant for and has received grant support from Angion BioMedica. RM is the Executive Editor of *KI Reports*.
